# Supplementary material for: Rare disease research workflow using multilayer networks elucidates the molecular determinants of severity in Congenital Myasthenic Syndromes
Source: Nat Commun. 2024 Feb 28;15:1227. doi: 10.1038/s41467-024-45099-0 (PMC10902324; doi:10.1038/s41467-024-45099-0)
Supplement: Supplementary file 1 — Supplementary Information [file 41467_2024_45099_MOESM1_ESM.pdf]

Supplementary Figures

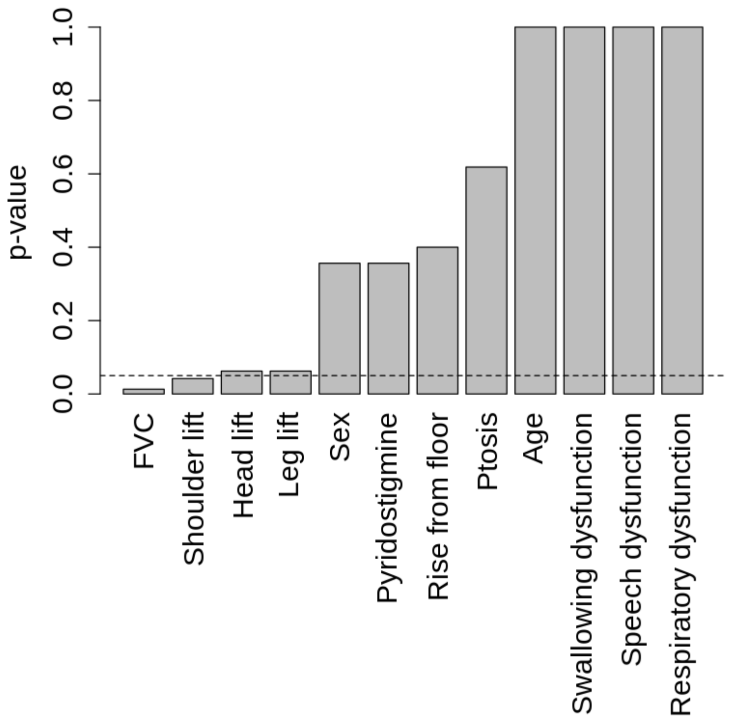

Suppl. Figure 1. Association between CMS severity (severe and not-severe phenotypes) and demographic factors (age, sex), pharmacological treatment (pyridostigmine), and clinical tests (speech, respiratory, swallowing functionality, ability to list shoulder, head, leg, eyelids (ptosis), and to rise from the floor, and Forced Vital Capacity (FVC)) (Suppl. Dataset 1). Classes were defined based on Suppl. Dataset 1. Age was discretized into two classes ('young' and 'old') based on the average age of all the patients (40 years). Barplot reports the p-values of a two-tailed Fisher's exact test (Methods). The dotted line indicates p = 0.05, n= 20 patients.

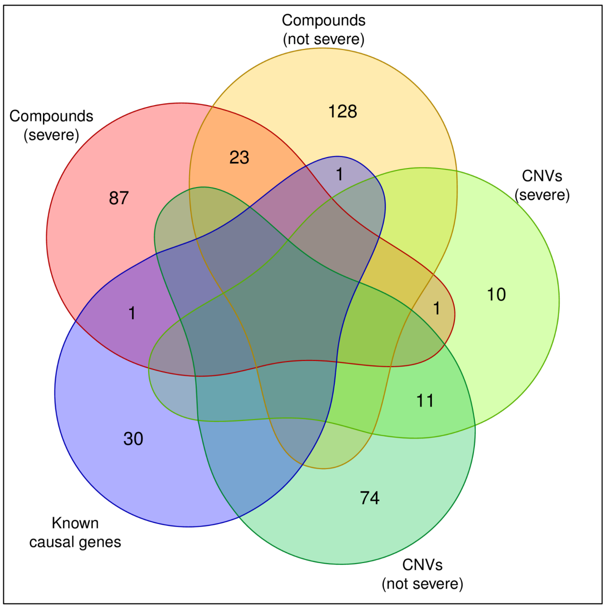

Suppl. Figure 2. Venn diagram of the genes associated with CNVs and compound heterozygous variants in not-severe and severe phenotypes as well as known CMS causal genes.

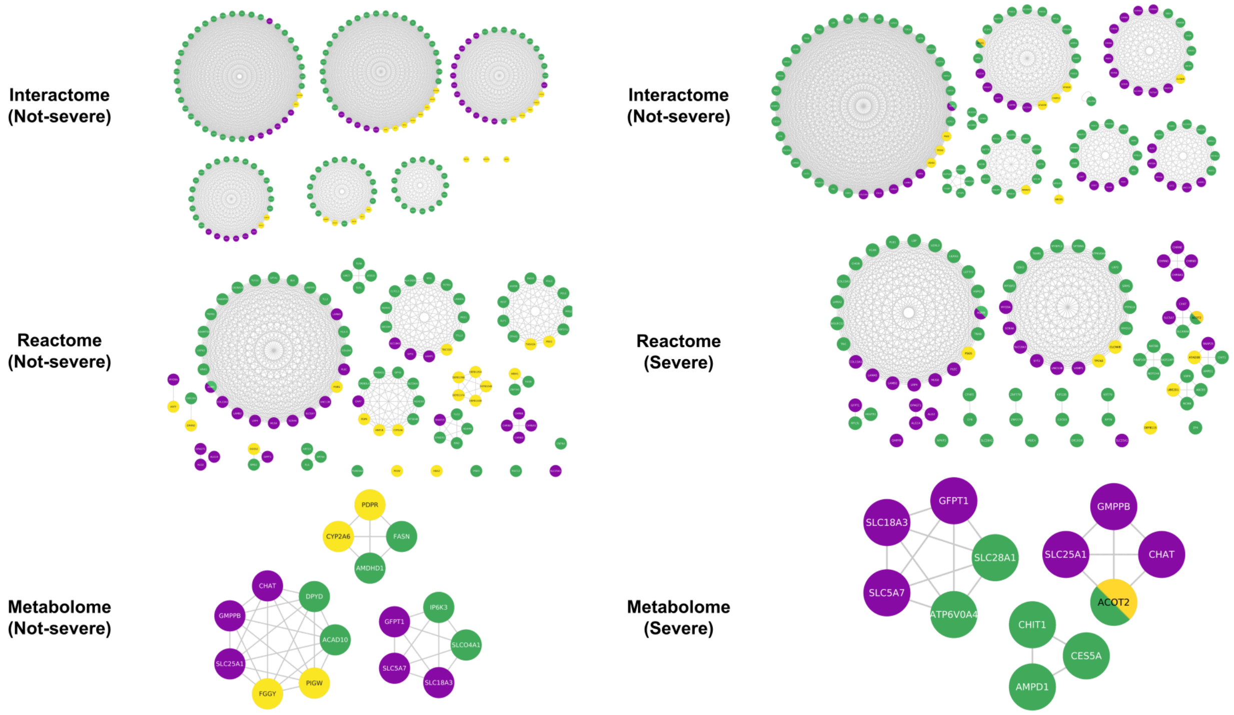

Suppl. Figure 3. Communities of CMS linked genes in the monolayer networks. Nodes are connected if they share membership to the same community from the clustering obtained using the Louvain algorithm. In green compound heterozygous variants; in yellow, CNVs; in purple, known CMS causal genes. Being a causal gene bearing compound heterozygous variants, AGRN is depicted in both purple and green. Being a gene presenting both compound heterozygous mutations and copy number variations, *ACOT2* is depicted in both green and yellow.

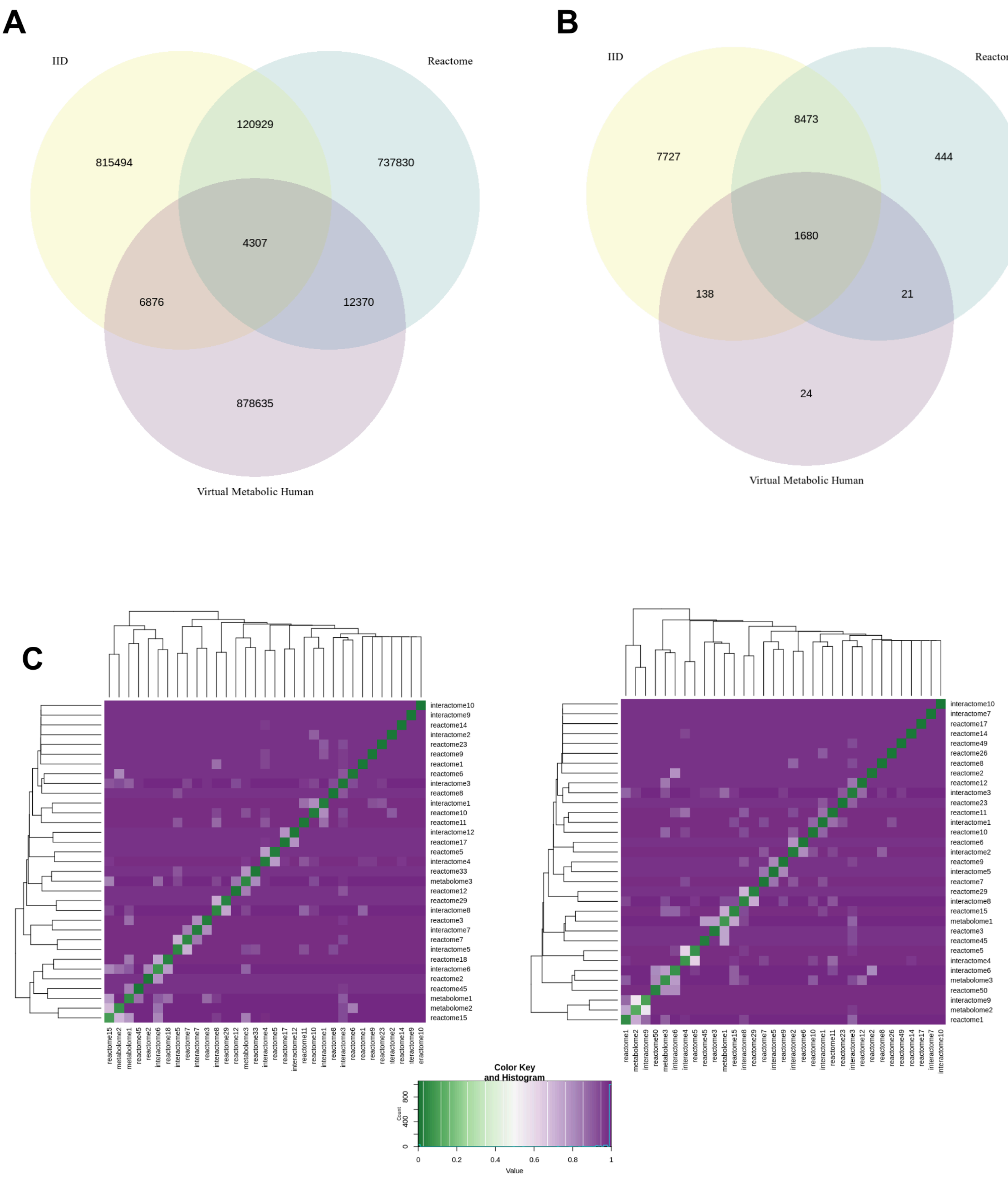

Suppl. Figure 4. (**A**) Edge overlap among the layers of the multilayer network. Each layer is identified by the name of the database from which the information was retrieved. (**B**) Node overlap among the layers of the multilayer network. (**C**) Heatmap of the Jaccard index dissimilarity among communities of CMS linked genes in the monolayer networks. Left, not-severe group, right, severe group.

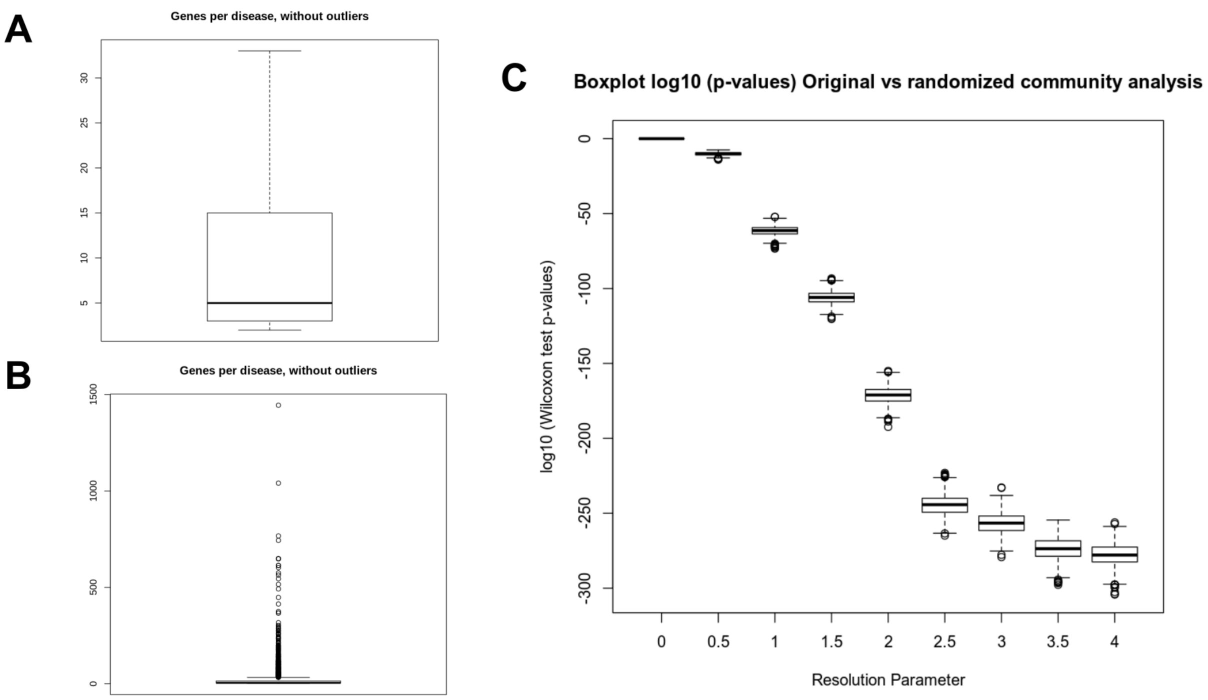

Suppl. Figure 5. Distribution of the number of genes per disease in the DisGeNET database, not showing (**A**) and showing (**B**) outliers (Methods) based on 1.5 * interquartile range criterion. Distribution of p-values (two-sided Wilcoxon test; logarithmic scale) associated with DisGeNET multilayer communities along the range of the MolTi resolution parameter under evaluation (**C**; Methods).

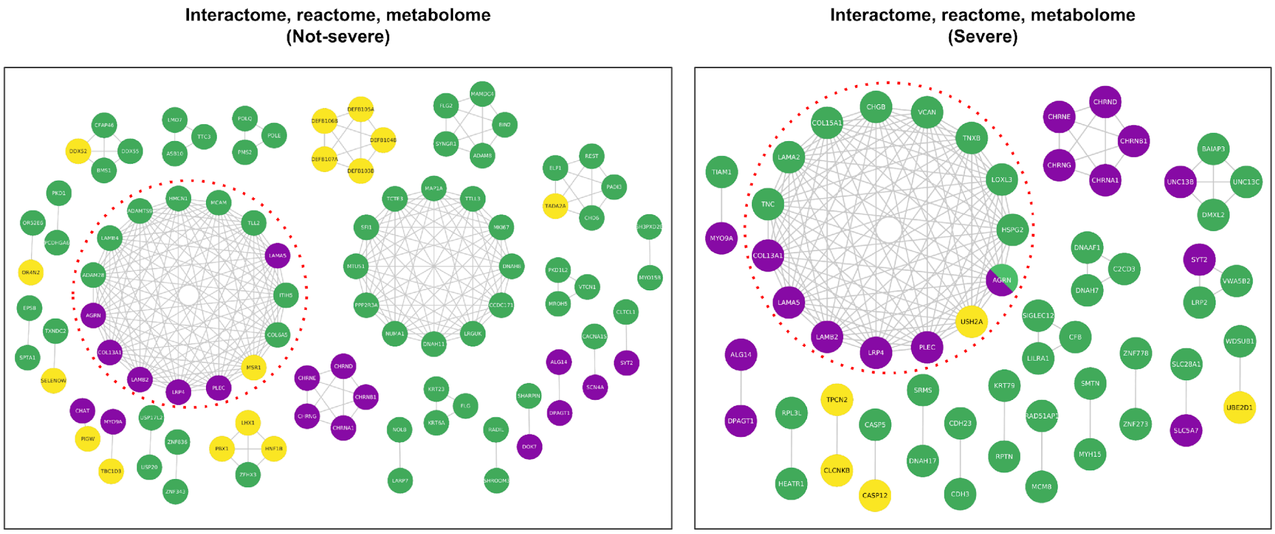

Suppl. Figure 6. Multilayer modules containing CMS linked genes of the severe and not-severe groups. Nodes are connected if the genes share membership to the same multilayer community across the range of MolTi resolution parameter (Methods). Modules with a size not expected to be found by chance are highlighted with a dotted circle (p-value < 0.05). In green compound heterozygous variants; in yellow, CNVs; in purple, known CMS causal genes.

Tissue Expression for AGRN (TPM)

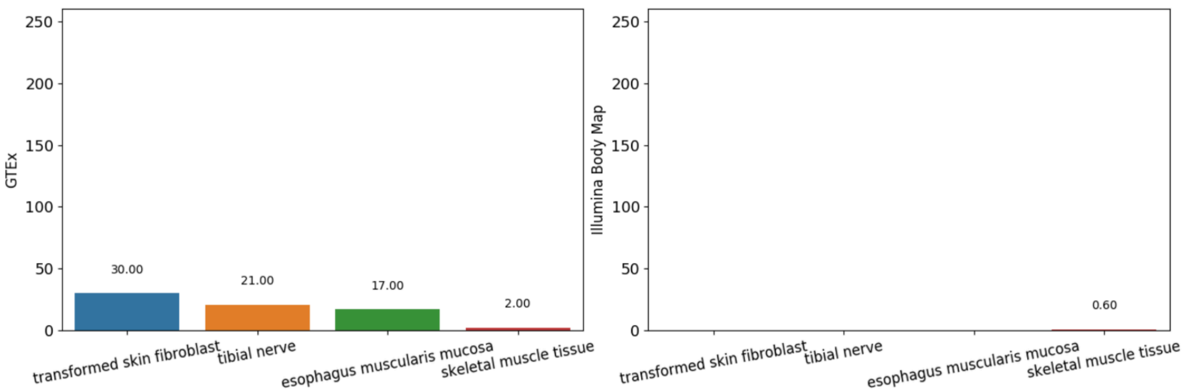

Tissue Expression for CHGB (TPM)

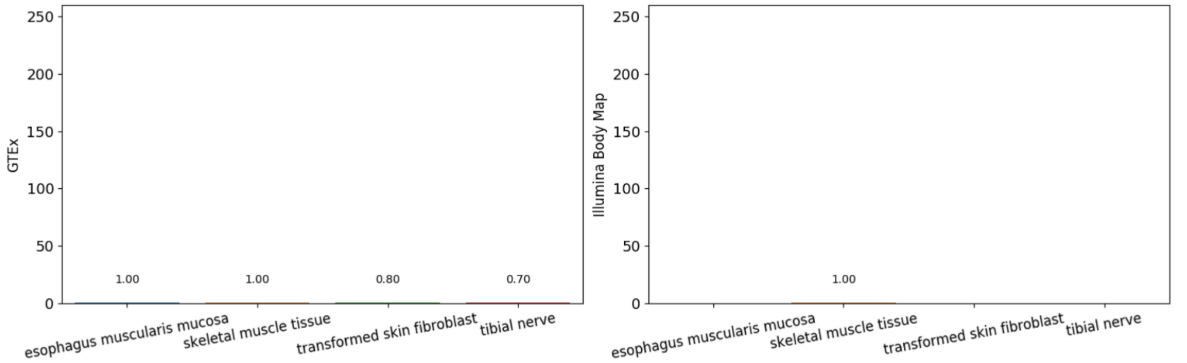

Tissue Expression for COL13A1 (TPM)

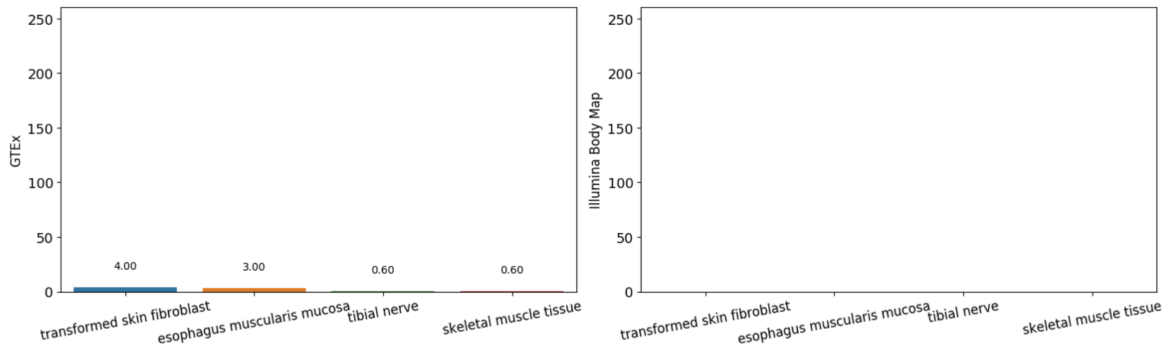

Tissue Expression for COL15A1 (TPM)

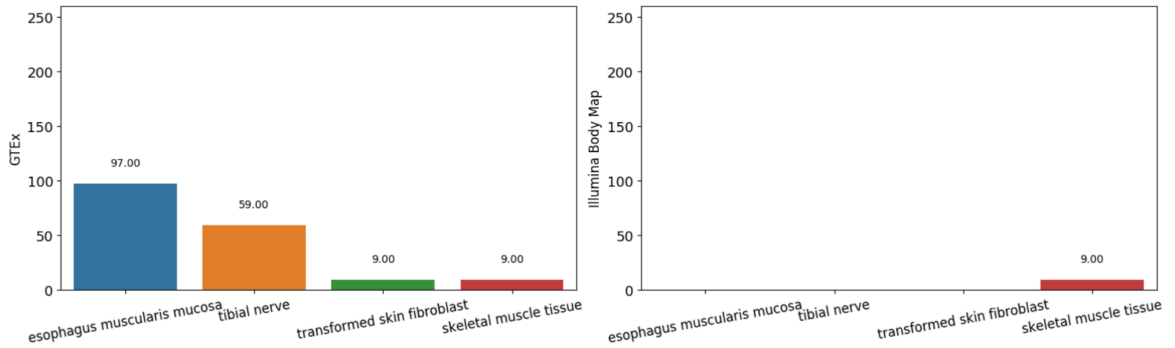

Tissue Expression for HSPG2 (TPM)

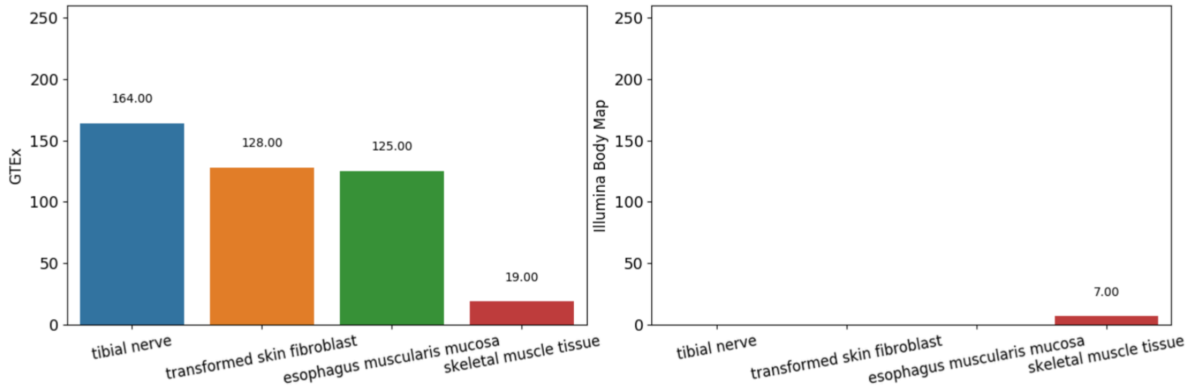

Tissue Expression for LAMA2 (TPM)

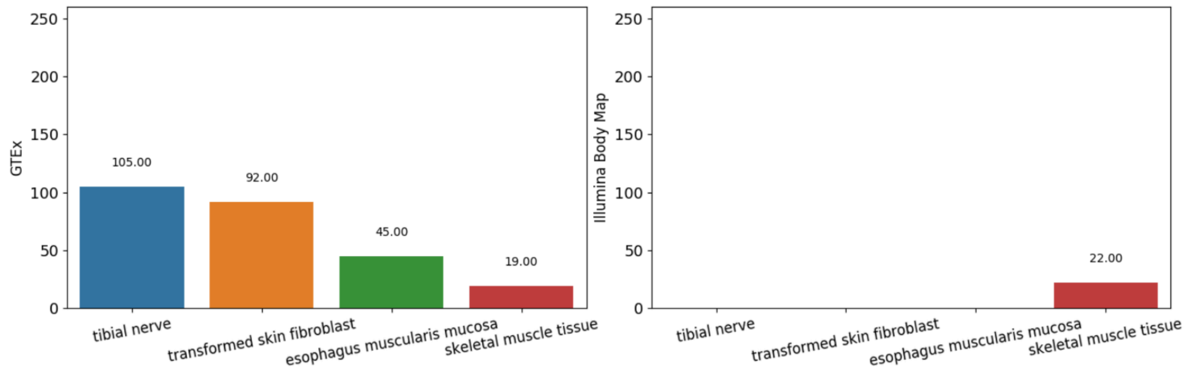

Tissue Expression for LAMA5 (TPM)

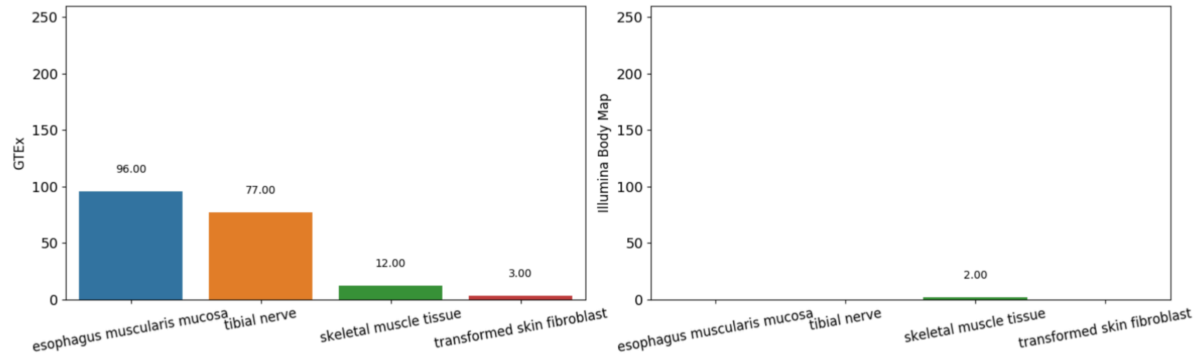

Tissue Expression for LAMB2 (TPM)

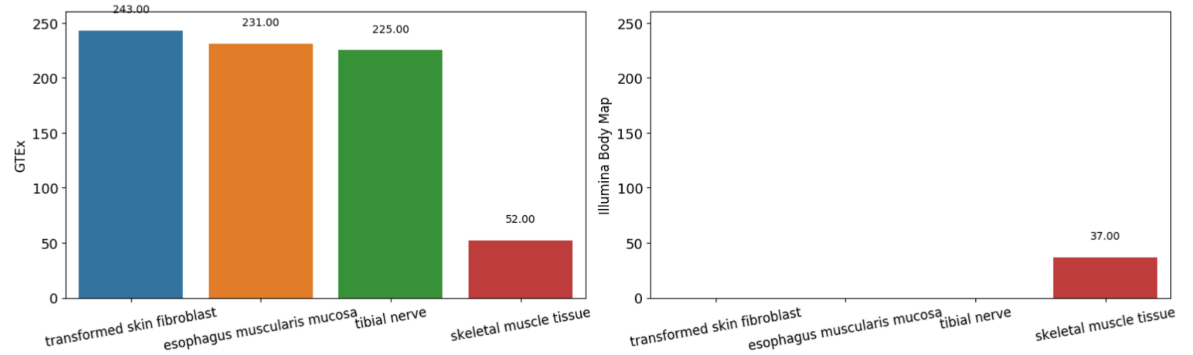

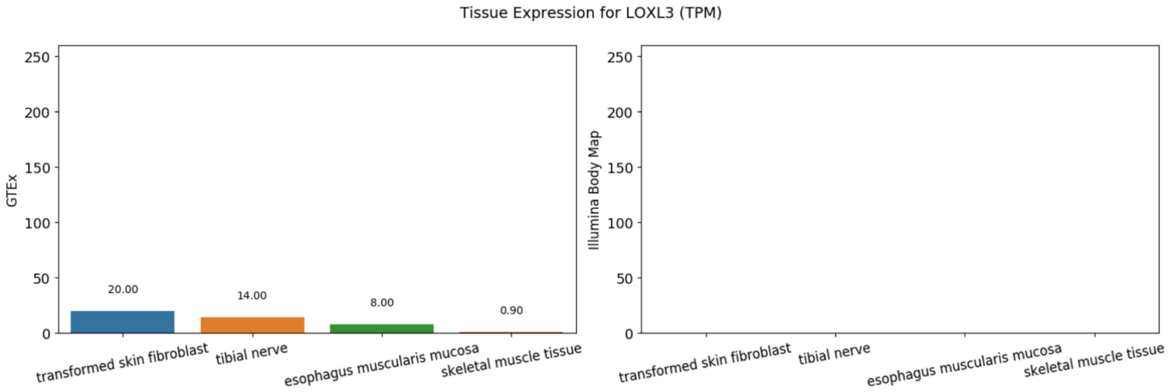
Tissue Expression for LOXL3 (TPM)
GTEx
Illumina Body Map
250
200
150
100
50
0
20.00
14.00
8.00
0.90
transformed skin fibroblast
tibial nerve
esophagus muscularis mucosa
skeletal muscle tissue

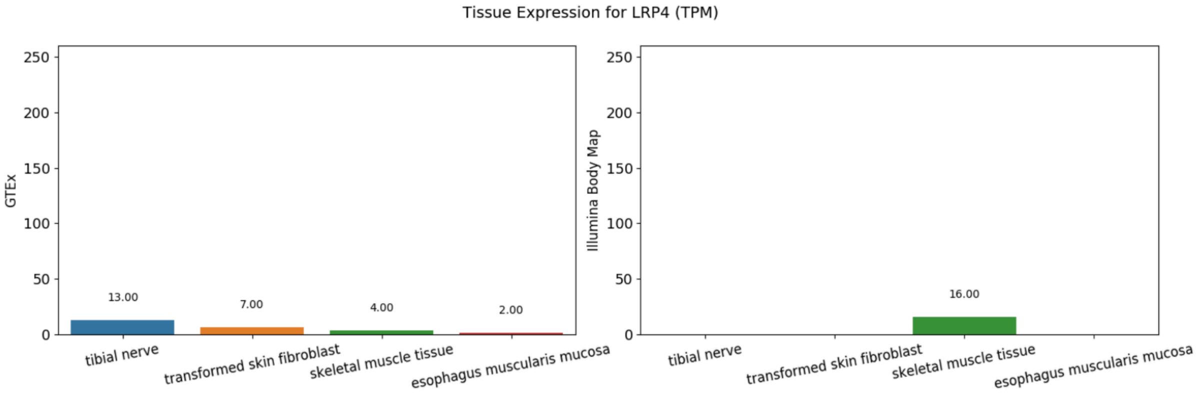
Tissue Expression for LRP4 (TPM)
GTEx
Illumina Body Map
250
200
150
100
50
0
13.00
7.00
4.00
2.00
16.00
tibial nerve
transformed skin fibroblast
skeletal muscle tissue
esophagus muscularis mucosa

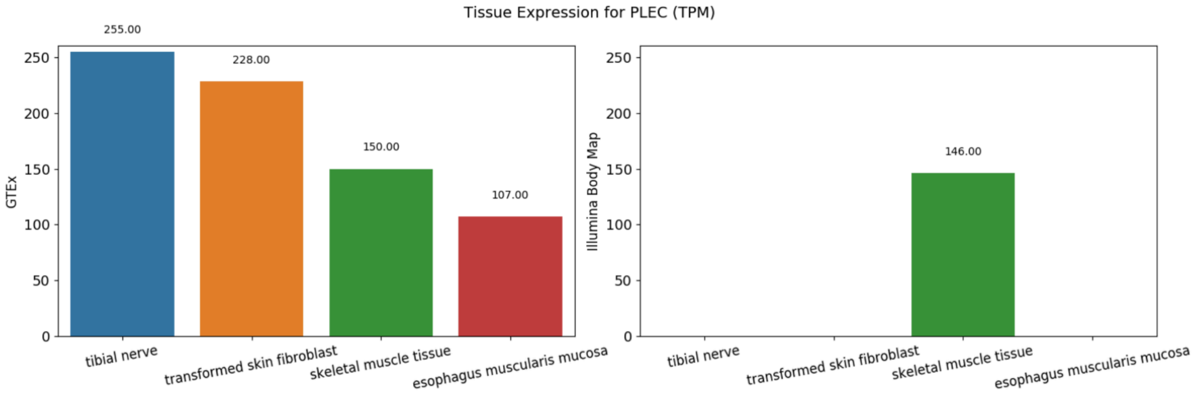
Tissue Expression for PLEC (TPM)
GTEx
Illumina Body Map
250
200
150
100
50
0
255.00
228.00
150.00
107.00
146.00
tibial nerve
transformed skin fibroblast
skeletal muscle tissue
esophagus muscularis mucosa

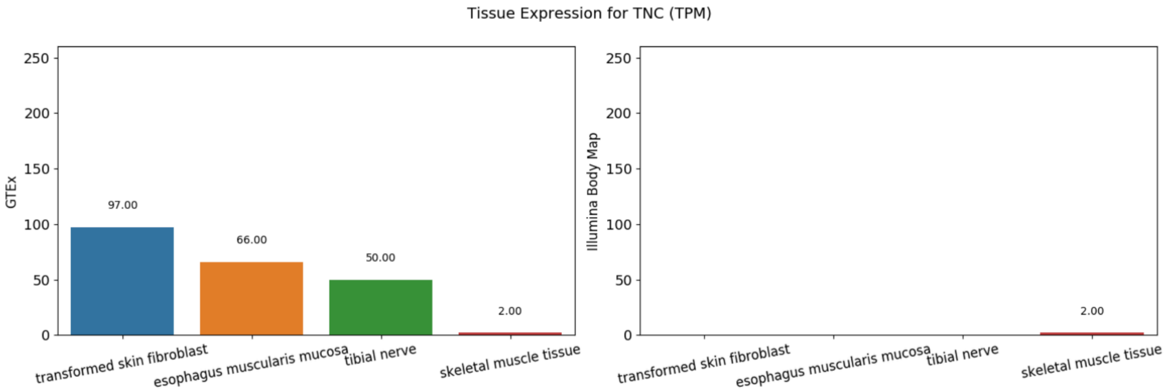
Tissue Expression for TNC (TPM)
GTEx
Illumina Body Map
250
200
150
100
50
0
97.00
66.00
50.00
2.00
2.00
transformed skin fibroblast
esophagus muscularis mucosa
tibial nerve
skeletal muscle tissue

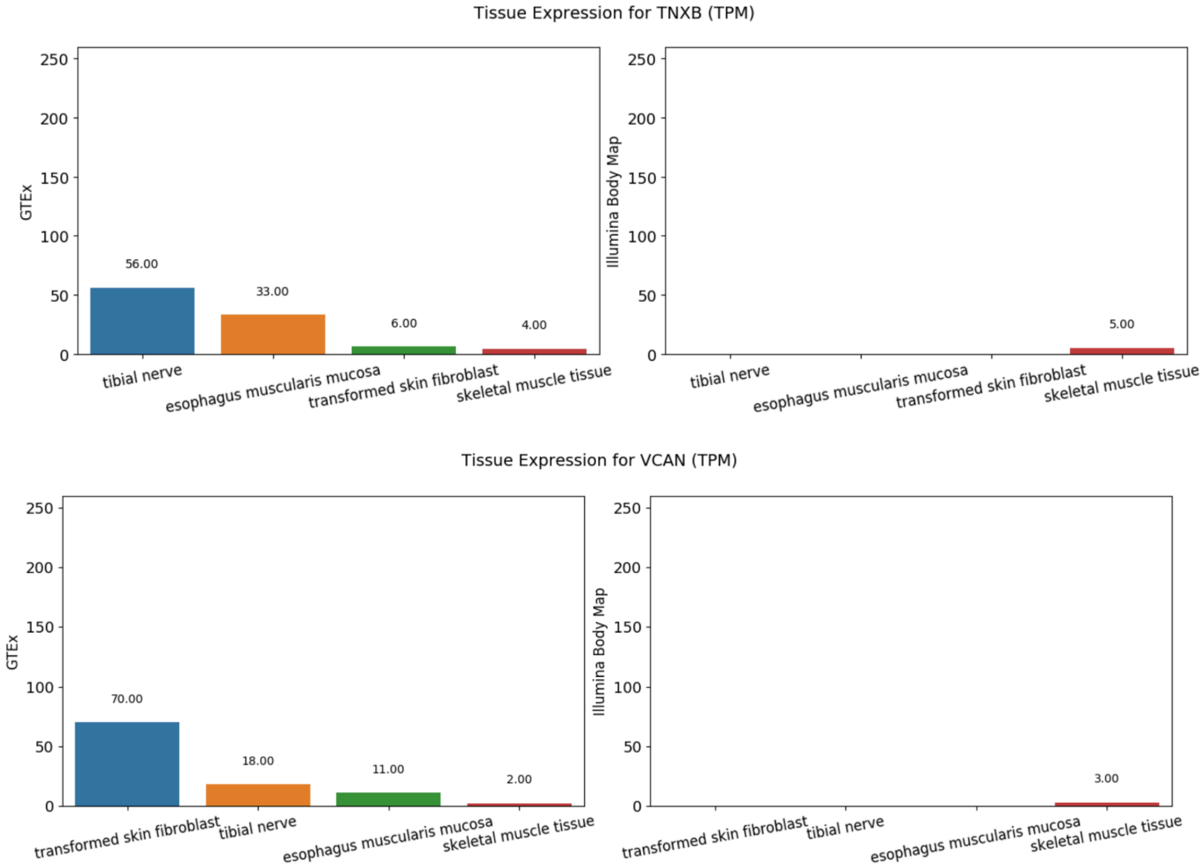

Suppl. Figure 7. Tissue-specific expression levels (Transcripts Per Million, TPM) of the genes contained in the largest module within the multilayer communities of the severe group (Methods). Expression levels are reported for GTEx (left panels) and Illumina Body Map (right panels) using EBI Expression Atlas default cutoff (0.5 TPM). Missing bars indicate no data availability (e.g. *COL13A1, LOXL3*). As its expression is below the cutoff for the tissues of interest in both GTEx and Illumina Body Map (Methods), *USH2A* is not reported. Expression level categories based on Expression Atlas: low (0.5 to 10 TPM), medium (11 to 1000 TPM), and high (more than 1000 TPM).

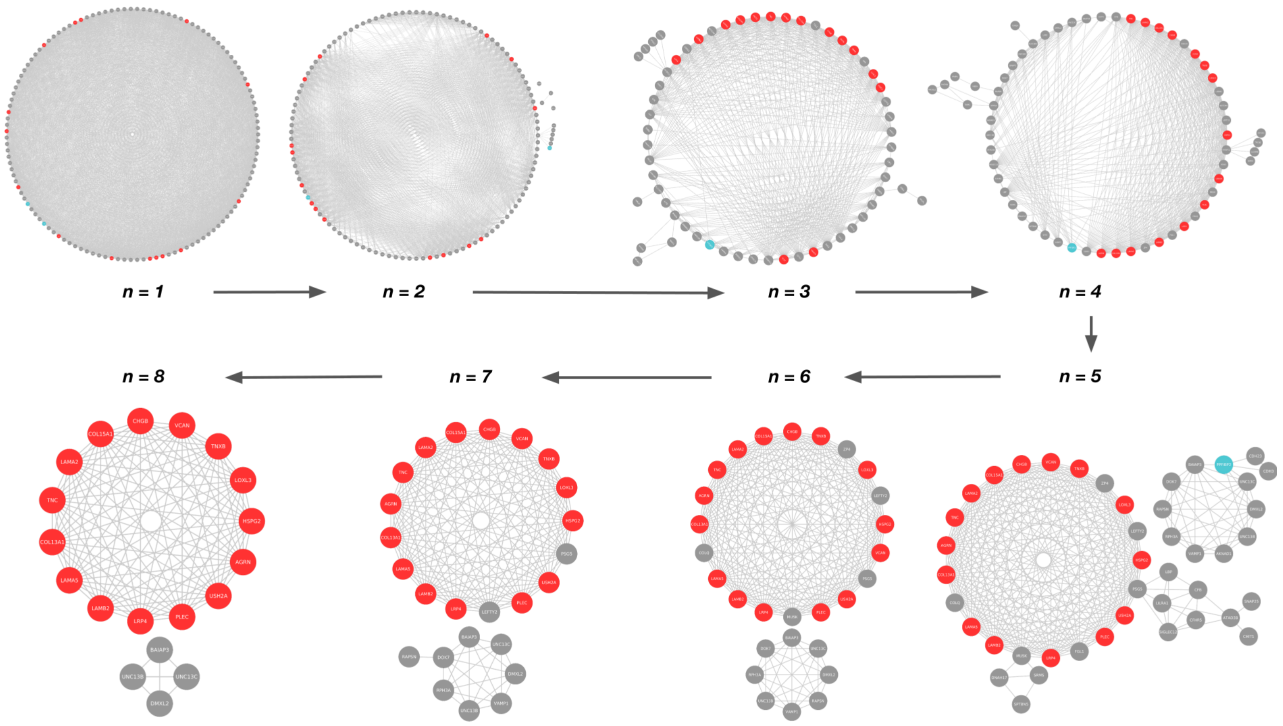

Suppl. Figure 8. Presence of *PPFIBP2* and *ACOT2* in the multilayer communities across the range of resolution parameter values (Methods). Genes of the severe-specific module are highlighted in red. *PPFIBP2* (present from n=1 to n=5) and *ACOT2* (present from n=1 to n=2) are depicted in blue.

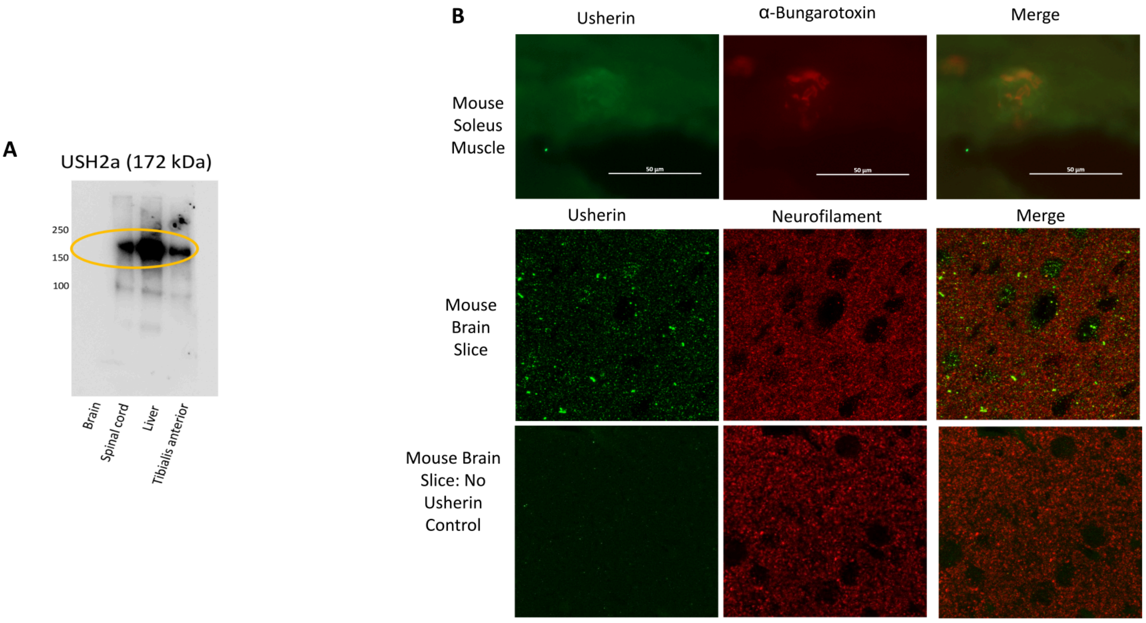

Suppl. Figure 9. Detection of Usherin in different tissues. (**A**) Western blot performed on 10-week-old mice detected a single band of the expected size in the spinal cord, liver, and tibialis anterior muscle, which is compatible with the ubiquitous expression of the protein in multiple tissues. Unfortunately, we were unable to detect the protein in the brain (Rb polyclonal IgG, FabGennix). (**B**) Using a directly conjugated Anti-Usherin-FITC antibody (Rb polyclonal, FabGennix) we were able to detect by immunofluorescence USH2A in mouse brain and at some neuromuscular junctions (α-Bungarotoxin) in mouse soleus muscle.

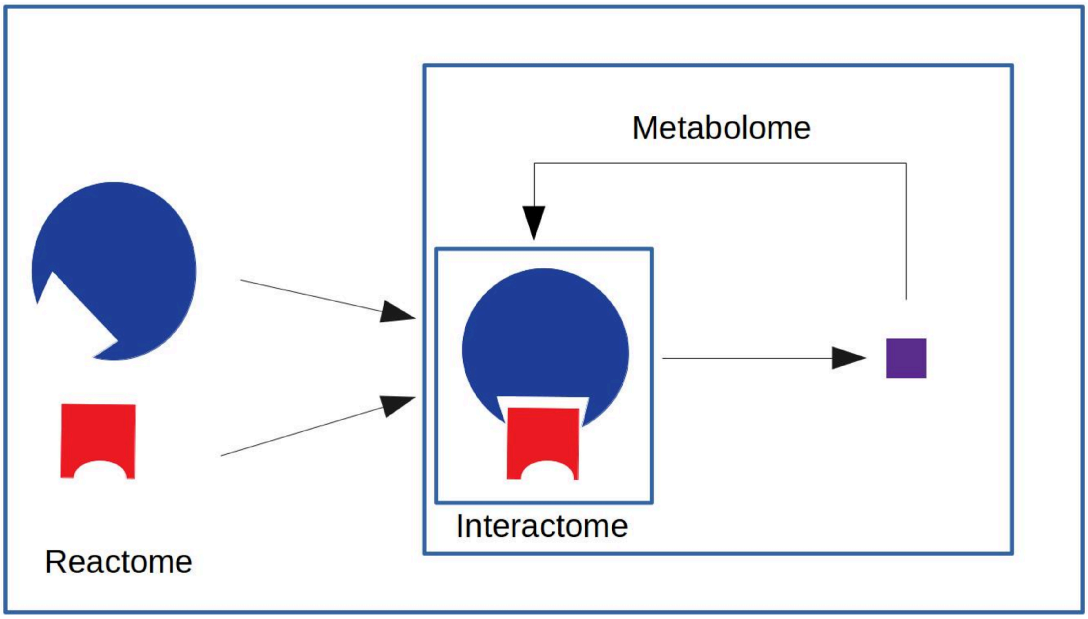

Suppl. Figure 10. Distinct layers of biological information covered in the analysis. In this example, enzyme A physically interacts (**interactome)** with enzyme B for the production of a metabolite that is further processed (**metabolome**). Moreover, all these molecules are part of the same pathway (**reactome**).

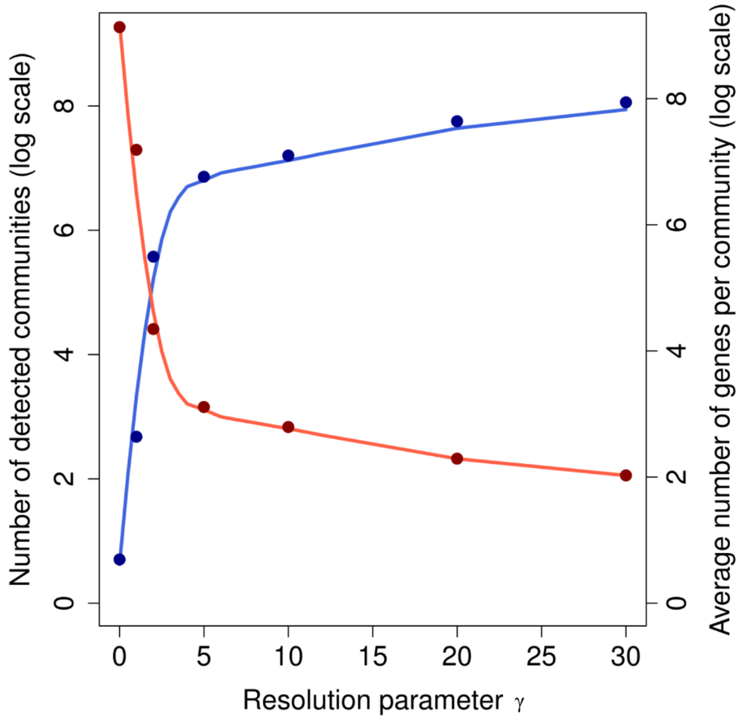

Suppl. Figure 11. Variation of number (blue line) and size (red line) of detected multilayer communities as a function of the MolTi resolution parameter γ. Curves are fitted with LOESS (locally estimated scatterplot smoothing) regression with span 0.6. Sampled points along the explored interval are shown for ease of visualization.

Appendix: Supplementary information

Functions of CMS-associated genes in the neuromuscular junction

Acetylcholine biosynthesis and release

Acetylcholine, the main neurotransmitter involved in skeletal muscle contraction, is synthesized in the presynaptic neuron, by the choline acetyltransferase (enzyme encoded by *CHAT* gene), using Acetyl-CoA and choline as substrate in the reaction [1]. Compound heterozygous mutations in this gene were identified by Ohno et al. [2] causing CMS in 5 patients. Solute carriers are critical for this process. Three genes encoding this class of transporters have been previously related to CMS and neuromuscular transmission defects, namely *SLC5A7* [3], *SLC25A* [4], and *SLC18A3* [5]. *SLC5A7* encodes the membrane choline transporter [6,7]. Acetyl-CoA presence is in part dependent on malate exported from mitochondria, by the action of *SLC25A1* transporter [8]. Finally, after *CHAT* generates the acetylcholine, this is carried into synaptic vesicles by *SLC18A3* gene product, the VAChT transporter [9]. Another CMS causal gene that might have a detrimental effect at presynaptic level is *PREPL*. This gene encodes a serine oligopeptidase essential for the activation of clathrin associated adaptor protein 1 (AP1), which is needed by VAChTr to fill the synaptic vesicles with acetylcholine [10] . Régal et al. [11] described a CMS case caused by a heterozygous deletion. Rabphilin 3a (*RPH3A*) is also involved in vesicle trafficking in the presynaptic element [12,13] and has recently been described as causative of a specific form of CMS [14]. Other genes described as causal of CMS related to the vesicle generation and exocytosis are *SNAP25* [15], *VAMP1* [16,17] , *SYT2* [18,19] and *UNC13B* [20].

SNAP25 encodes synaptosomal-associated protein 25 [21], which is a part of the SNARE complex, where also synaptobrevin 1 (*VAMP1*) is allocated [22]. This SNARE complex is key for the Ca^{2+}-induced exocytosis of synaptic vesicles, a process in which Synaptotagmin 2 (*SYT2*), the Ca2+ sensor, is also critical [23]. *UNC13B* encodes a homolog protein to rat Munc13-1. This protein has a calmodulin site and also regulates synaptic vesicles by mediating in the SNARE complex conformation [24].

Acetylcholine Receptor clustering

While acetylcholine is the main neurotransmitter in the neuromuscular junction contraction process, another important molecule, the proteoglycan agrin (*AGRN*), is released by exocytosis from the motor neuron into the synaptic cleft, where it binds the *LRP4* receptor. A special type of myosin, *MYO9*, is known to affect *AGRN* exocytosis upon depletion, causing a characteristic type of CMS [25,26]. *AGRN* binding to *LRP4* leads to MuSK (*MUSK*) self-phosphorylation. Activated MuSK recruits Dok-7 (*DOK7*), which in the end stimulates Rapsyn (*RAPSN*) for AChRs (acetylcholine receptors) clustering at the skeletal muscle fiber membrane [27] . MuSK, Dok-7 and Rapsyn absence has been previously reported to result in AChR deficiency, poor neuromuscular junction development and causal of some CMS cases [28–31]. Interestingly, promoting MuSK activity has been described as capable of preserving neuromuscular synapses in Amyotrophic Lateral Sclerosis mice models [32]. Plectin, encoded by the gene *PLEC*, is essential in the AChR clustering process as it bridges AChRs to the postsynaptic intermediate filament network (IF) via interaction with rapsyn [33]. Mutations in this gene are also described to cause CMS [34,35]. MuSK is also required for the

anchoring of endplate acetylcholinesterase (AChE) at the NMJ extracellular matrix (ECM), via a collagen-like peptide encoded by *COLQ* gene [36]. AChE is involved in terminating impulse transmission, by hydrolysis of acetylcholine. Mutations in *COLQ* have been reported as causative for a specific form of CMS [37,38].

The acetylcholine receptor itself is the main source of CMS-related mutations. In adult individuals, the receptor acts as a cation ligand-gated ion channel formed by 5 homologous subunits, being α2βδε its stoichiometry. The channel is mainly permeable to Na^+ and K^+, and to Ca^{2+} in a lesser way. When acetylcholine binds to AChR, the channel opens triggering the membrane depolarization [39]. All the genes encoding the receptor subunits (*CHRNA, CHRNB, CHRND, CHRNE* and *CHRNG*) have been described as causal for different CMS types [40–44]. *CHRNE*, which encodes the ε subunit of the AChR receptor, is causative for ~50% of all reported CMS cases, although frequencies might vary depending on ethnicity [45,46]. The high prevalence of ε subunit mutations may be the result of partial compensation of its functionality by the embryonic γ (encoded by *CHRNG*), which is substituted after birth given its lower conductance levels. Mutations in other subunits reduce patient survival as no compensation mechanism occurs [47]. Both Fast-Channel CMS (abnormally short AChR opening time) and Slow-Channel (abnormally long AChR opening time) CMS have been reported for mutations on *CHRNE.*

SCN4A gene encodes the α subunit of the voltage-gated sodium channel ($Na_v1.4$), which is key for the generation and propagation of action potentials through the skeletal muscle fiber, which causes Ca^{2+} release and fiber contraction. Many *SCN4A* mutations have been associated with different muscle channelopathies [48–50], including CMS.

Another process related to AChR clustering is the Endoplasmic Reticulum glycosylation pathway. Normally, mutations in genes that are part of these processes cause congenital disorders of glycosylation (CDG) [51]. However, mutations in some of the pathways components (*DPAGT1*, *ALG2*, *ALG14* , *GFPT1* and *GMPPB*) have been also described as causal of some CMS variants [52–55]. As for the ECM, collagens are also involved in the receptor clustering process. Collagen XIII (encoded by COL13A1 gene) is known to be a key regulator of NMJ maturation process and AChR clustering [56]. Logan et al. [57] reported a specific form of CMS being caused by mutations on this gene. Laminins α5 and β2 are also involved molecules in AChR clustering [58]. Each one of the different laminins have its own role during NMJ maturation and development, with mutations in LAMA5 [59] and LAMB2 [60] being causative of the CMS disease.

Segregation analyses

We employed Rbbt [61] framework to stratify CMS patients based on mutations, aiming to assess whether nonsevere (n=12) and severe (n=8) patients segregate any of the following mutation types (Figure S1):

'**overlapping**' = the mutation overlaps the span of the gene, from first exon to last, including introns
'**mutated_isoform**' = the mutation produces a mutated isoform, i.e. an AA change (on one isoform or just the principal isoform, depending on the options used)
'**splicing**' = the mutation falls within a splicing site, they are deemed to break the protein function
'**affected**' = the mutations affects the encoded protein, by introducing a mutated isoform or a splice site mutation

'damaged_mutated_isoform' = the mutation makes a specific protein isoform damaged as predicted by damage or pathogenicity predictions
'broken' = the mutation seems to break the protein function, due it introducing a damaging mutation or a splice site mutation
'TSS' = the mutation falls within a transcription starting site (1000 bases from TSS)
'compound' = the gene has at least two mutations that affect it
'homozygous' = the gene is affected by a homozygous mutation
'missing' = the genes function may be entirely missing due to a homozygous or a compound mutation possibly affecting both alleles
'gc19_pc' .promCore' = core promoter of protein coding gene (hg19)
'gc19_pc.promDomain' = promoter domain of protein coding gene (hg19)
'gc19_pc.5utr' = 5'UTR of a protein coding gene (hg19)
'gc19_pc.3utr' = 3'UTR of a protein coding gene (hg19)
'gc19_pc.ss' = splicing site of a protein coding gene (hg19)
'lncrna.promDomain' = core promoter of a long noncoding RNA with coding potential
'lncrna.promCore' = core promoter of a long noncoding RNA with coding potential
'lncrna.ss' = splicing site of a long noncoding RNA with coding potential
'lncrna.ncrna' = long noncoding RNA
'smallrna.ncrna' = small RNA

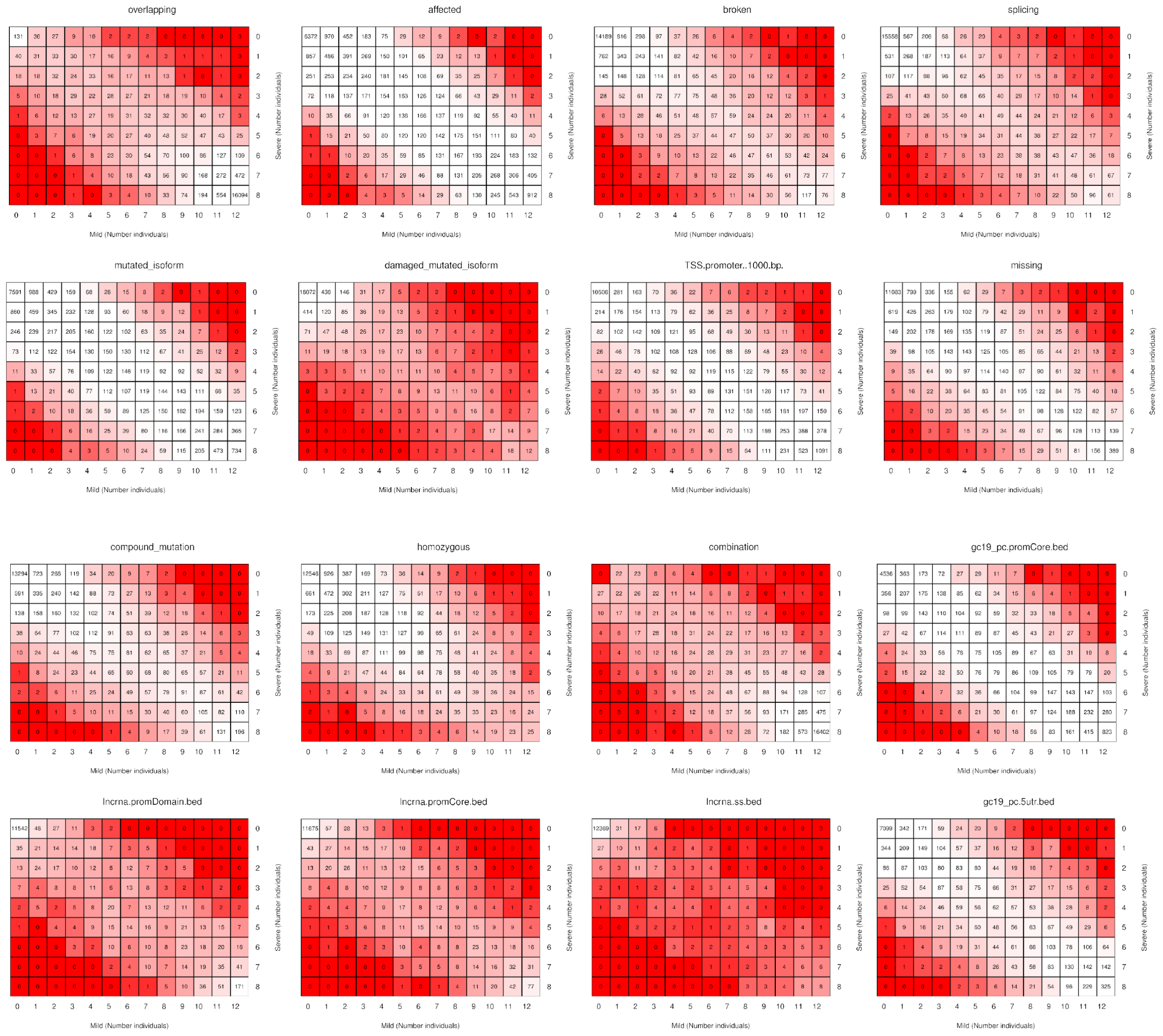

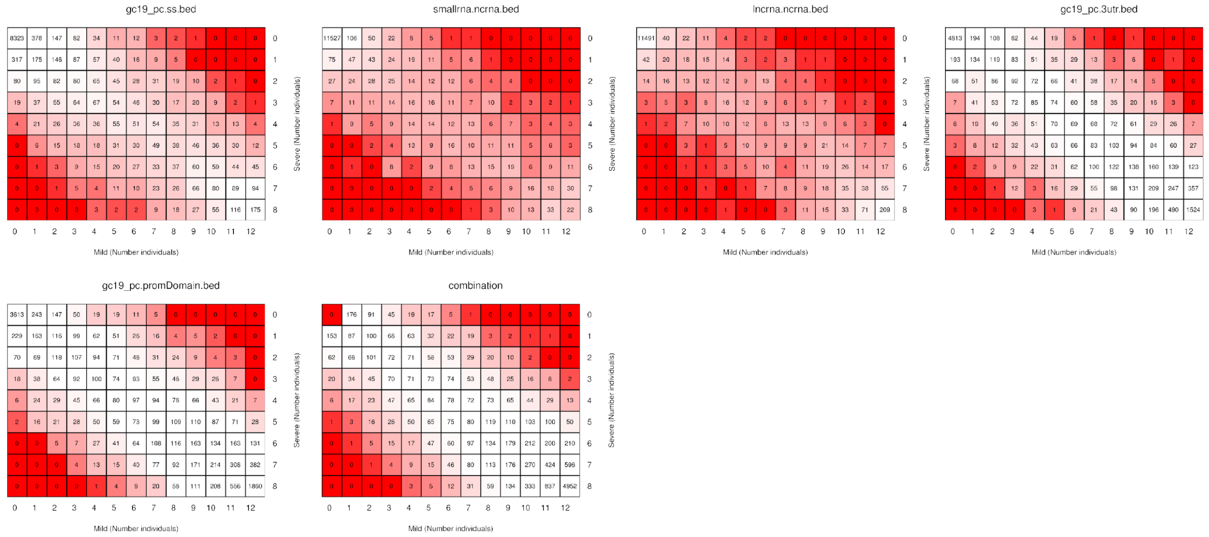

Figure S1. Segregation analysis of several mutation types (described in the text). The number of mutations that overlap in the two groups (Nonsevere and Severe) are reported for sets of individuals (0 to 12 for nonsevere individuals, 0 to 8 for severe individuals).

We define complete segregating mutations that are present in one group and not in the other. No complete segregating mutations were observed (Figure S1), while partial segregation mutations (i.e. present in at least 50% of the patients of one group and not in the other) can be appreciated (Suppl. Dataset 2).

Multilayer community detection analysis

In this work, we performed a multilayer community detection analysis using MolTi software [62], which adapts the Louvain clustering algorithm with modularity maximization to multilayer networks. The algorithm is parametrized by the resolution parameter γ: the higher the value of γ, the smaller the size of the detected multilayer communities.
Given the intrinsic resolution limit of modularity, the reliability of community detection should be assessed *ad hoc* using quality functions that are able to capture the actual community structure of a network [63]. In this work, we were interested in the identification of communities that robustly express functional relationships among the CMS linked genes (i.e. known CMS causal genes, and severe and nonsevere compound heterozygous variants and CNVs). Accordingly, we sought to determine the largest module of CMS linked genes that are found in the same multilayer communities at any value of resolution within the parameter range in which the community structure is more variable (see Supplementary Figure 12). The adopted procedure is illustrated in Figure S2.

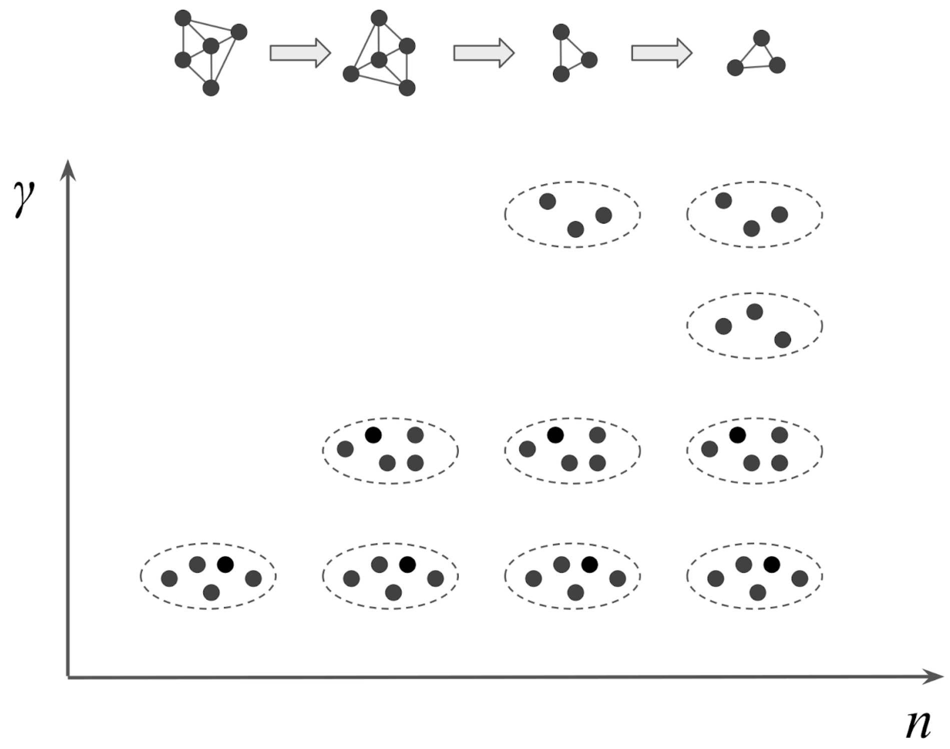

Figure S2. Module identification based on detected multilayer communities. Genes that are found in the same community at *n* values of the resolution parameter γ are represented as fully connected modules (upper panel). The resolution range considered is $\gamma \in (0,4]$ with intervals of 0.5 (Methods). The module corresponding to the highest *n* contains genes that are systematically found in the same community across the entire range of resolution.

Supplementary Information References

1. Nachmansohn, D. & Machado, A. L. The formation of acetylcholine. a new enzyme: 'choline acetylase'. *J. Neurophysiol.* **6**, 397–403 (1943).
2. Ohno, K. *et al.* Choline acetyltransferase mutations cause myasthenic syndrome associated with episodic apnea in humans. *Proc. Natl. Acad. Sci.* **98**, 2017–2022 (2001).
3. Bauché, S. *et al.* Impaired Presynaptic High-Affinity Choline Transporter Causes a Congenital Myasthenic Syndrome with Episodic Apnea. *Am. J. Hum. Genet.* **99**, 753–761 (2016).
4. Chaouch, A. *et al.* Mutations in the Mitochondrial Citrate Carrier SLC25A1 are Associated with Impaired Neuromuscular Transmission. *J. Neuromuscul. Dis.* **1**, 75–90 (2014).
5. O'Grady, G. L. *et al.* Variants in SLC18A3, vesicular acetylcholine transporter, cause congenital myasthenic syndrome. *Neurology* **87**, 1442–1448 (2016).
6. Okuda, T. & Haga, T. Functional characterization of the human high-affinity choline transporter 1. *FEBS Lett.* **484**, 92–97 (2000).
7. Apparsundaram, S., Ferguson, S. M. & Blakely, R. D. Molecular cloning and characterization of a murine hemicholinium-3-sensitive choline transporter. *Biochem. Soc. Trans.* **29**, 711–716 (2001).
8. Kaplan, R. S., Mayor, J. A. & Wood, D. O. The mitochondrial tricarboxylate transport protein. cDNA cloning, primary structure, and comparison with other mitochondrial transport proteins.

J. Biol. Chem. **268**, 13682–13690 (1993).
9. Eiden, L. E., Schäfer, M. K.-H., Weihe, E. & Schütz, B. The vesicular amine transporter family (SLC18): amine/proton antiporters required for vesicular accumulation and regulated exocytotic secretion of monoamines and acetylcholine. *Pflugers Arch.* **447**, 636–640 (2004).
10. Radhakrishnan, K., Baltes, J., Creemers, J. W. M. & Schu, P. Trans-Golgi network morphology and sorting is regulated by prolyl-oligopeptidase-like protein PREPL and the AP-1 complex subunit µ1A. *J. Cell Sci.* **126**, 1155–1163 (2013).
11. Régal, L. *et al.* PREPL deficiency with or without cystinuria causes a novel myasthenic syndrome. *Neurology* **82**, 1254–1260 (2014).
12. Guillén, J. *et al.* Structural insights into the Ca2+ and PI(4,5)P2 binding modes of the C2 domains of rabphilin 3A and synaptotagmin 1. *Proc. Natl. Acad. Sci.* **110**, 20503–20508 (2013).
13. Shirataki, H. *et al.* Rabphilin-3A, a putative target protein for smg p25A/rab3A p25 small GTP-binding protein related to synaptotagmin. *Mol. Cell. Biol.* **13**, 2061–2068 (1993).
14. Maselli, R. A. *et al.* Presynaptic congenital myasthenic syndrome with altered synaptic vesicle homeostasis linked to compound heterozygous sequence variants in RPH3A. *Mol. Genet. Genomic Med.* **6**, 434–440 (2018).
15. Shen, X.-M., Selcen, D., Brengman, J. & Engel, A. G. Mutant SNAP25B causes myasthenia, cortical hyperexcitability, ataxia, and intellectual disability. *Neurology* **83**, 2247–2255 (2014).
16. Salpietro, V. *et al.* Homozygous mutations in VAMP1 cause a presynaptic congenital myasthenic syndrome. *Ann. Neurol.* **81**, 597–603 (2017).
17. Shen, X.-M. *et al.* Novel synaptobrevin-1 mutation causes fatal congenital myasthenic syndrome. *Ann. Clin. Transl. Neurol.* **4**, 130–138 (2017).
18. Herrmann, D. N. *et al.* Synaptotagmin 2 Mutations Cause an Autosomal-Dominant Form of Lambert-Eaton Myasthenic Syndrome and Nonprogressive Motor Neuropathy. *Am. J. Hum. Genet.* **95**, 332–339 (2014).
19. Whittaker, R. G. *et al.* Electrophysiologic features of SYT2 mutations causing a treatable neuromuscular syndrome. *Neurology* **85**, 1964–1971 (2015).
20. Engel, A. G., Selcen, D., Shen, X.-M., Milone, M. & Harper, C. M. Loss of MUNC13-1 function causes microcephaly, cortical hyperexcitability, and fatal myasthenia. *Neurol. Genet.* **2**, e105 (2016).
21. Sørensen, J. B. *et al.* Differential Control of the Releasable Vesicle Pools by SNAP-25 Splice Variants and SNAP-23. *Cell* **114**, 75–86 (2003).
22. Liu, Y., Sugiura, Y. & Lin, W. The role of Synaptobrevin1/VAMP1 in Ca2+-triggered neurotransmitter release at the mouse neuromuscular junction. *J. Physiol.* **589**, 1603–1618 (2011).
23. Pang, Z. P., Sun, J., Rizo, J., Maximov, A. & Südhof, T. C. Genetic analysis of synaptotagmin 2 in spontaneous and Ca2+-triggered neurotransmitter release. *EMBO J.* **25**, 2039–2050 (2006).
24. Ma, C., Li, W., Xu, Y. & Rizo, J. Munc13 mediates the transition from the closed syntaxin–Munc18 complex to the SNARE complex. *Nat. Struct. Mol. Biol.* **18**, 542–549 (2011).
25. O'Connor, E. *et al.* Identification of mutations in the MYO9A gene in patients with congenital myasthenic syndrome. *Brain* **139**, 2143–2153 (2016).
26. O'Connor, E. *et al.* MYO9A deficiency in motor neurons is associated with reduced neuromuscular agrin secretion. *Hum. Mol. Genet.* **27**, 1434–1446 (2018).
27. Burden, S. J., Yumoto, N. & Zhang, W. The Role of MuSK in Synapse Formation and Neuromuscular Disease. *Cold Spring Harb. Perspect. Biol.* **5**, a009167 (2013).
28. Chevessier, F. *et al.* MUSK, a new target for mutations causing congenital myasthenic

syndrome. *Hum. Mol. Genet.* **13**, 3229–3240 (2004).
29. Azulay, J. P. *et al.* [Isolated proximal muscular weakness disclosing myasthenic syndrome]. *Rev. Neurol. (Paris)* **150**, 377–381 (1994).
30. Ohno, K. *et al.* Rapsyn Mutations in Humans Cause Endplate Acetylcholine-Receptor Deficiency and Myasthenic Syndrome. *Am. J. Hum. Genet.* **70**, 875–885 (2002).
31. Kumar, A., Asghar, S., Kavanagh, R. & Wicklund, M. P. Unique presentation of rapidly fluctuating symptoms in a child with congenital myasthenic syndrome due to RAPSN mutation. *Muscle Nerve* **58**, E23–E24 (2018).
32. Cantor, S. *et al.* Preserving neuromuscular synapses in ALS by stimulating MuSK with a therapeutic agonist antibody. *eLife* **7**, e34375 (2018).
33. Mihailovska, E. *et al.* Neuromuscular synapse integrity requires linkage of acetylcholine receptors to postsynaptic intermediate filament networks via rapsyn–plectin 1f complexes. *Mol. Biol. Cell* **25**, 4130–4149 (2014).
34. Banwell, B. L. *et al.* Myopathy, myasthenic syndrome, and epidermolysis bullosa simplex due to plectin deficiency. *J. Neuropathol. Exp. Neurol.* **58**, 832–846 (1999).
35. Selcen, D. *et al.* Myasthenic syndrome caused by plectinopathy. *Neurology* **76**, 327–336 (2011).
36. Cartaud, A. *et al.* MuSK is required for anchoring acetylcholinesterase at the neuromuscular junction. *J. Cell Biol.* **165**, 505–515 (2004).
37. Ohno, K., Brengman, J., Tsujino, A. & Engel, A. G. Human endplate acetylcholinesterase deficiency caused by mutations in the collagen-like tail subunit (ColQ) of the asymmetric enzyme. *Proc. Natl. Acad. Sci.* **95**, 9654–9659 (1998).
38. Donger, C. *et al.* Mutation in the human acetylcholinesterase-associated collagen gene, COLQ, is responsible for congenital myasthenic syndrome with end-plate acetylcholinesterase deficiency (Type Ic). *Am. J. Hum. Genet.* **63**, 967–975 (1998).
39. Brisson, A. & Unwin, P. N. T. Quaternary structure of the acetylcholine receptor. *Nature* **315**, 474–477 (1985).
40. Engel, A. G. *et al.* A newly recognized congenital myasthenic syndrome attributed to a prolonged open time of the acetylcholine-induced ion channel. *Ann. Neurol.* **11**, 553–569 (1982).
41. Quiram, P. A. *et al.* Mutation causing congenital myasthenia reveals acetylcholine receptor β/δ subunit interaction essential for assembly. *J. Clin. Invest.* **104**, 1403–1410 (1999).
42. Brownlow, S. *et al.* Acetylcholine receptor δ subunit mutations underlie a fast-channel myasthenic syndrome and arthrogryposis multiplex congenita. *J. Clin. Invest.* **108**, 125–130 (2001).
43. Ohno, K. *et al.* Congenital myasthenic syndrome caused by prolonged acetylcholine receptor channel openings due to a mutation in the M2 domain of the epsilon subunit. *Proc. Natl. Acad. Sci.* **92**, 758–762 (1995).
44. Morgan, N. V. *et al.* Mutations in the Embryonal Subunit of the Acetylcholine Receptor (CHRNG) Cause Lethal and Escobar Variants of Multiple Pterygium Syndrome. *Am. J. Hum. Genet.* **79**, 390–395 (2006).
45. Abicht, A., Müller, J. S. & Lochmüller, H. Congenital Myasthenic Syndromes Overview. in *GeneReviews®* (eds. Adam, M. P. et al.) (University of Washington, Seattle, 1993).
46. Finsterer, J. Congenital myasthenic syndromes. *Orphanet J. Rare Dis.* **14**, 57 (2019).
47. Engel, A. G., Ohno, K., Bouzat, C., Sine, S. M. & Griggs, R. C. End-plate acetylcholine receptor deficiency due to nonsense mutations in the epsilon subunit. *Ann. Neurol.* **40**, 810–817 (1996).

48. Wu, F., Mi, W., Fu, Y., Struyk, A. & Cannon, S. C. Mice with an Na V 1.4 sodium channel null allele have latent myasthenia, without susceptibility to periodic paralysis. *Brain* **139**, 1688–1699 (2016).
49. Zaharieva, I. T. *et al.* Loss-of-function mutations in SCN4A cause severe foetal hypokinesia or 'classical' congenital myopathy. *Brain* **139**, 674–691 (2016).
50. Tsujino, A. *et al.* Myasthenic syndrome caused by mutation of the SCN4A sodium channel. *Proc. Natl. Acad. Sci.* **100**, 7377–7382 (2003).
51. Jaeken, J. & Matthijs, G. From glycosylation to glycosylation diseases. *Biochim. Biophys. Acta BBA - Mol. Basis Dis.* **1792**, 823 (2009).
52. Belaya, K. *et al.* Mutations in DPAGT1 Cause a Limb-Girdle Congenital Myasthenic Syndrome with Tubular Aggregates. *Am. J. Hum. Genet.* **91**, 193–201 (2012).
53. Belaya, K. *et al.* Mutations in GMPPB cause congenital myasthenic syndrome and bridge myasthenic disorders with dystroglycanopathies. *Brain* **138**, 2493–2504 (2015).
54. Cossins, J. *et al.* Congenital myasthenic syndromes due to mutations in ALG2 and ALG14. *Brain J. Neurol.* **136**, 944–956 (2013).
55. Senderek, J. *et al.* Hexosamine Biosynthetic Pathway Mutations Cause Neuromuscular Transmission Defect. *Am. J. Hum. Genet.* **88**, 162–172 (2011).
56. Latvanlehto, A. *et al.* Muscle-Derived Collagen XIII Regulates Maturation of the Skeletal Neuromuscular Junction. *J. Neurosci.* **30**, 12230–12241 (2010).
57. Logan, C. V. *et al.* Congenital Myasthenic Syndrome Type 19 Is Caused by Mutations in COL13A1, Encoding the Atypical Non-fibrillar Collagen Type XIII α1 Chain. *Am. J. Hum. Genet.* **97**, 878–885 (2015).
58. Rogers, R. S. & Nishimune, H. The role of laminins in the organization and function of neuromuscular junctions. *Matrix Biol.* **57–58**, 86–105 (2017).
59. Maselli, R. A. *et al.* Presynaptic congenital myasthenic syndrome with a homozygous sequence variant in LAMA5 combines myopia, facial tics, and failure of neuromuscular transmission. *Am. J. Med. Genet. A.* **173**, 2240–2245 (2017).
60. Maselli, R. A. *et al.* Mutations in LAMB2 causing a severe form of synaptic congenital myasthenic syndrome. *J. Med. Genet.* **46**, 203–208 (2009).
61. Vázquez, M., Nogales, R., Carmona, P., Pascual, A. & Pavón, J. Rbbt: A Framework for Fast Bioinformatics Development with Ruby. in *Advances in Bioinformatics* (eds. Rocha, M. P., Riverola, F. F., Shatkay, H. & Corchado, J. M.) 201–208 (Springer, 2010). doi:10.1007/978-3-642-13214-8_26.
62. Didier, G., Brun, C. & Baudot, A. Identifying communities from multiplex biological networks. *PeerJ* **2015**, (2015).
63. Fortunato, S. & Barthélemy, M. Resolution limit in community detection. *Proc. Natl. Acad. Sci.* **104**, 36–41 (2007).
