# Supplementary material for: Rare disease research workflow using multilayer networks elucidates the molecular determinants of severity in Congenital Myasthenic Syndromes
Source: Nat Commun. 2024 Feb 28;15:1227. doi: 10.1038/s41467-024-45099-0 (PMC10902324; doi:10.1038/s41467-024-45099-0)
Supplement: Supplementary file 3 — Description of Additional Supplementary Files [file 41467_2024_45099_MOESM3_ESM.pdf]

Description of Additional Supplementary Files

Supplementary Datasets legends

Suppl. Dataset 1. Clinical characterization of 20 CMS patients with distinct severity levels, namely severe and not-severe (mild and moderate). The annotation of clinical test responses with Human Phenotype Ontology (HPO) (https://hpo.jax.org/) terms has been manually curated. FCV: Forced Vital Capacity, i.e. volume of air that can forcibly be blown out after full inspiration. Y: yes. N: no. NI: no information.

Suppl. Dataset 2. Partially segregating mutations. In the table, mutations segregating at least 50% of one group (i.e. 5 out of 8 severe and 6 out of 10 mild patients) are reported (the mutation categories are described in Supplementary Information, Segregation analyses).

Suppl. Dataset 3. Genes associated with CNVs and compound heterozygous variants in not-severe and severe phenotypes. Severe-specific genes and known CMS causal genes are reported.

Suppl. Dataset 4. Estimated familiar relatedness between the analyzed patients. Only patients presenting positive relatedness (Methods) are shown.

Suppl. Dataset 5. Functional effect prediction of Ensembl VEP (Method) for the compound heterozygous variants found in the largest module within the multilayer communities of the severe group. Deleterious variants are highlighted in bold.

Suppl. Dataset 6. Functional effect prediction of Ensembl VEP (Method) for the compound heterozygous variants found in Patient 3. Deleterious variants are highlighted in bold.
