# Supplementary material for: Rare disease research workflow using multilayer networks elucidates the molecular determinants of severity in Congenital Myasthenic Syndromes
Source: Nat Commun. 2024 Feb 28;15:1227. doi: 10.1038/s41467-024-45099-0 (PMC10902324; doi:10.1038/s41467-024-45099-0)
Supplement: Supplementary file 5 — Reporting Summary [file 41467_2024_45099_MOESM5_ESM.pdf]

nature portfolio

Corresponding author(s): Davide Cirillo

Last updated by author(s): Dec 19, 2023

Reporting Summary

Nature Portfolio wishes to improve the reproducibility of the work that we publish. This form provides structure for consistency and transparency in reporting. For further information on Nature Portfolio policies, see our Editorial Policies and the Editorial Policy Checklist.

Statistics

For all statistical analyses, confirm that the following items are present in the figure legend, table legend, main text, or Methods section.

n/a	Confirmed	
☐	☒	The exact sample size (*n*) for each experimental group/condition, given as a discrete number and unit of measurement
☒	☐	A statement on whether measurements were taken from distinct samples or whether the same sample was measured repeatedly
☐	☒	The statistical test(s) used AND whether they are one- or two-sided<br>*Only common tests should be described solely by name; describe more complex techniques in the Methods section.*
☐	☒	A description of all covariates tested
☐	☒	A description of any assumptions or corrections, such as tests of normality and adjustment for multiple comparisons
☐	☒	A full description of the statistical parameters including central tendency (e.g. means) or other basic estimates (e.g. regression coefficient) AND variation (e.g. standard deviation) or associated estimates of uncertainty (e.g. confidence intervals)
☐	☒	For null hypothesis testing, the test statistic (e.g. F, t, r) with confidence intervals, effect sizes, degrees of freedom and P value noted<br>*Give P values as exact values whenever suitable.*
☒	☐	For Bayesian analysis, information on the choice of priors and Markov chain Monte Carlo settings
☒	☐	For hierarchical and complex designs, identification of the appropriate level for tests and full reporting of outcomes
☒	☐	Estimates of effect sizes (e.g. Cohen's *d*, Pearson's *r*), indicating how they were calculated

Our web collection on statistics for biologists contains articles on many of the points above.

Software and code

Policy information about availability of computer code

Data collection	WGS data was preprocessed applying the RD-Connect specific pipeline using BWA-mem for alignment and Picard for duplicate marking and GATK 3.6.0 for variant calling. RNA-seq data was preprocessed using STAR 2.35a for alignment, RSEM 1.3.0 for quantification, and GATK 3.6.0 for variant calling. CNV data was preprocessed using ClinCNV (https://github.com/imgag/ClinCNV). Compound heterozygous variants data was preprocessed using phASER (Castel et al. 2016), EAGLE2 (Durbin 2014), and eDiVA (Bosio et al. 2019).
Data analysis	All code and the Cytoscape session rendering Figures 3 and 4, as well as Supplementary Figures 3, 6 and 9 are available for reproducibility purposes at: https://github.com/ikernunezca/CMS. The analysis of multilayer communities can also be performed using CmmD (https://github.com/ikernunezca/CmmD) with parameters: resolution_start: 0, resolution_end: 4, interval: 0.5 and the CMS linked genes as nodelist. Code can also be referenced using Zenodo (doi:10.5281/zenodo.10352689).

For manuscripts utilizing custom algorithms or software that are central to the research but not yet described in published literature, software must be made available to editors and reviewers. We strongly encourage code deposition in a community repository (e.g. GitHub). See the Nature Portfolio guidelines for submitting code & software for further information.

Data

Policy information about availability of data

All manuscripts must include a data availability statement. This statement should provide the following information, where applicable:

- Accession codes, unique identifiers, or web links for publicly available datasets
- A description of any restrictions on data availability
- For clinical datasets or third party data, please ensure that the statement adheres to our policy

WGS metadata and variant data, and patient phenotypic descriptions have been deposited in the RD-Connect GPAP: https://platform.rd-connect.eu/#/. This data is available are under controlled access in the Data for registered users of the GPAP. Details on access can be found in: https://platform.rd-connect.eu/userregistration. Biobank sample accession identifiers are provided in Supplementary Table 1. The raw RNA-Seq dataset analyzed in this study is not publicly available due to sensible content (patient molecular data on a rare disease). Minimal, pre-processed RNA-Seq data for reproducibility is provided within the github repository of the project: CMS/data/fibroblast_expression/. Reasonable requests for further information will be carefully evaluated by the corresponding author and co-authors. All Source Data files for plotting are provided within the github repository of the project: https://github.com/ikernunezca/CMS. Information on the source data can be accessed from : https://github.com/ikernunezca/CMS/blob/master/Source_Information_README.

We specifically provide the Cytoscape Session file ('.cys') containing all the plots used to produce Figures 3 and 4, as well as Supplementary Figures 3, 6, and 8 in this link:
https://github.com/ikernunezca/CMS/blob/master/Cytoscape_Session/CMS_Session.cys
Specific input Source Data files for creating the Cytoscape Session used to build Figure 3, 4A, 4B, 6 and 8 can be accessed from the following link as csv files:
https://github.com/ikernunezca/CMS/tree/master/Cytoscape_Session
Additionally, the Cytoscape Session provides an extra plot with the incident interactions considered to render Figure 5. Supplementary Figure 1 source data is provided as Supplementary Table 1. Input Data for reproducing Supplementary Figure 2 can be accessed from: https://github.com/ikernunezca/CMS/tree/master/data/InputGenes. Input for plotting Supplementary Figure 11 as well as information on the files is available at: https://github.com/ikernunezca/CMS/tree/master/data/MolTi/Community_Analysis.

We carried out the multilayer network analysis using three (monolayer) networks, obtained from Reactome database 32, from the Recon3D Virtual Metabolic Human database 33 (both downloaded in May 2018), and from the Integrated Interaction Database (IID) 34 (downloaded in October 2018).

Research involving human participants, their data, or biological material

Policy information about studies with human participants or human data. See also policy information about sex, gender (identity/presentation), and sexual orientation and race, ethnicity and racism.

Reporting on sex and gender	Sex (biological attribute) is considered in the study and is reported in Suppl. Table 1.
Reporting on race, ethnicity, or other socially relevant groupings	We report the ethnicity of the cohort within the manuscript.
Population characteristics	Demographic and phenotypic characteristics are reported in Suppl. Table 1.
Recruitment	Determination of phenotypes of interest for the study was performed by expert clinicians (I.T. and V.G.), taking a detailed medical history and performing a full neurological exam.
Ethics oversight	This study was approved by the Ethics committee of Sofia Medical University (protocol 4/15-April-2013)

Note that full information on the approval of the study protocol must also be provided in the manuscript.

Field-specific reporting

Please select the one below that is the best fit for your research. If you are not sure, read the appropriate sections before making your selection.

☒ Life sciences ☐ Behavioural & social sciences ☐ Ecological, evolutionary & environmental sciences

For a reference copy of the document with all sections, see nature.com/documents/nr-reporting-summary-flat.pdf

Life sciences study design

All studies must disclose on these points even when the disclosure is negative.

Sample size	The study focuses on a rare disease cohort for which samples of 20 patients were available.
Data exclusions	No data were excluded from the analyses.
Replication	No replicates were available as they are precious rare biological human samples and cannot be analysed in replicates.

Randomization	Randomization was not possible as the study focuses on a rare disease cohort for which samples of 20 patients were available.
Blinding	The investigators were unblinded to group allocation during human data collection.

Reporting for specific materials, systems and methods

We require information from authors about some types of materials, experimental systems and methods used in many studies. Here, indicate whether each material, system or method listed is relevant to your study. If you are not sure if a list item applies to your research, read the appropriate section before selecting a response.

Materials & experimental systems

n/a	Involved in the study
☐	☒ Antibodies
☒	☐ Eukaryotic cell lines
☒	☐ Palaeontology and archaeology
☐	☒ Animals and other organisms
☒	☐ Clinical data
☒	☐ Dual use research of concern
☒	☐ Plants

Methods

n/a	Involved in the study
☒	☐ ChIP-seq
☒	☐ Flow cytometry
☒	☐ MRI-based neuroimaging

Antibodies

Antibodies used	For Western Blotting 40mg of protein was run on a 10% gel and transferred to a membrane using the BioRad Trans Turbo semi-dry transfer machine. The membrane was blocked in milk for 1 hour and Usherin (FabGennix, USH2A-112AP, 1:2000) was added (5% BSA in TBST) overnight. Secondary antibodies were diluted 1:1000 in milk.<br>Labelling of the neuromuscular junction (NMJ) was performed on soleus muscle. Muscles were washed in ice-cold PBS (2 x 10 mins) and then separated out into small bundles under a stereo-microscope. They were fixed overnight at 4°C in 2% PFA, washed 2 x 1hr with ice-cold PBS, and treated with Analar Ethanol and Methanol both at -20°C (10mins each). Tissues were then incubated with blocking/permeabilization solution (5% horse serum, 5% BSA, 2% Triton X-100 in PBS) for 4 hours (room temp (RT)) with gentle agitation. Muscle bundles were incubated with antibodies, diluted in blocking buffer without triton, against Usherin-FITC (Rb polyclonal, FabGennix USH.101-FITC, 1:100) overnight (4°C) with agitation and then for a further 2 hrs (RT) the following morning. Muscles were then washed in blocking buffer 4 x 1 hr (RT) and incubated with Alexa 594-Conjugated α-Bungarotoxin (ThermoFisherScientific, B13423, 1:250), for 4 hrs (RT). Samples were washed 4 x 1 hr in PBS and then mounted using Vectashield hardset mounting medium. Images were captured using Olympus FV1000c scanning confocal microscope using FV1000 application software (FV10-ASW) software at x63 oil immersion objective.
Validation	Manufacturer information about the used antibodies can be found at the following web sites: https://fabgennix.com/Usherin-Antibody-USH-112AP and https://fabgennix.com/Usherin-Antibody-FITC.

Animals and other research organisms

Policy information about studies involving animals; ARRIVE guidelines recommended for reporting animal research, and Sex and Gender in Research

Laboratory animals	10-week-old C57BL/6J (Jax) male mice were housed under 12h light/dark cycles and had ad libitum access to standard chow (Teklad Global 18% protein Rodent Diet) and water.
Wild animals	Not wild animal.
Reporting on sex	Sex was not considered in animal study design because no sex differences were observed in human (Suppl. Figure 1).
Field-collected samples	No field collected samples were used in the study.
Ethics oversight	All animal experiments were approved by the University of Ottawa animal care and veterinary service department (protocol #3089) and complied with the guidelines of the Canadian Council on Animal Care and the Animals for Research Act.

Note that full information on the approval of the study protocol must also be provided in the manuscript.
